# Peroxisomes contribute to reactive oxygen species homeostasis and cell division induction in *Arabidopsis* protoplasts

**DOI:** 10.3389/fpls.2015.00658

**Published:** 2015-08-26

**Authors:** Terence W.-Y. Tiew, Michael B. Sheahan, Ray J. Rose

**Affiliations:** School of Environmental and Life Sciences, The University of Newcastle, CallaghanNSW, Australia

**Keywords:** *Arabidopsis*, cell division, cytoskeleton, peroxisomes, *Nicotiana*, protoplasts, reactive oxygen species, totipotency

## Abstract

The ability to induce *Arabidopsis* protoplasts to dedifferentiate and divide provides a convenient system to analyze organelle dynamics in plant cells acquiring totipotency. Using peroxisome-targeted fluorescent proteins, we show that during protoplast culture, peroxisomes undergo massive proliferation and disperse uniformly around the cell before cell division. Peroxisome dispersion is influenced by the cytoskeleton, ensuring unbiased segregation during cell division. Considering their role in oxidative metabolism, we also investigated how peroxisomes influence homeostasis of reactive oxygen species (ROS). Protoplast isolation induces an oxidative burst, with mitochondria the likely major ROS producers. Subsequently ROS levels in protoplast cultures decline, correlating with the increase in peroxisomes, suggesting that peroxisome proliferation may also aid restoration of ROS homeostasis. Transcriptional profiling showed up-regulation of several peroxisome-localized antioxidant enzymes, most notably catalase (CAT). Analysis of antioxidant levels, CAT activity and CAT isoform 3 mutants (*cat3*) indicate that peroxisome-localized CAT plays a major role in restoring ROS homeostasis. Furthermore, protoplast cultures of *pex11a*, a peroxisome division mutant, and *cat3* mutants show reduced induction of cell division. Taken together, the data indicate that peroxisome proliferation and CAT contribute to ROS homeostasis and subsequent protoplast division induction.

## Introduction

Plant protoplasts provide an invaluable experimental system to study cellular processes such as signal transduction (Sheen, 2001), cell wall regeneration (Leucci et al., 2007), the role of stress and hormones (Pasternak et al., 2002) and transient gene expression (Yoo et al., 2007). Protoplasts are well suited for investigating organelles in plant cells, as they provide many cytological benefits not available to multicellular tissues and suspension-cultured cells that commonly exist in clusters. More importantly, differentiated protoplasts can be induced to acquire totipotency and regenerate (Takebe et al., 1971; Chupeau et al., 2013), providing a convenient system to analyze organelle dynamics and inheritance in plant cells. In this system we have previously examined chloroplasts, the endoplasmic reticulum (ER; Sheahan et al., 2004), mitochondria (Sheahan et al., 2004, 2005), and vacuoles (Sheahan et al., 2007a); particularly in relation to the cytoskeleton. These studies have provided insights into the proliferation and inheritance of these organelles and the identification of “massive mitochondrial fusion.” Given the significance of peroxisomes in plant metabolism (del Rio et al., 2006; Palma et al., 2009; Hu et al., 2012), we have investigated the dynamics of peroxisomes in cultured protoplasts acquiring totipotency in relation to proliferation, inheritance and reactive oxygen species (ROS) homeostasis.

Peroxisomes are ubiquitous in most eukaryotic cells (Baker and Graham, 2002) and in plant cells, are essential for the oxidation of fatty acids, photorespiration, biosynthesis of hormones and in ROS metabolism (Beevers, 1979; Johnson and Olsen, 2001; Hayashi and Nishimura, 2003; Nyathi and Baker, 2006; Hu et al., 2012). Peroxisomes are metabolically plastic organelles which adapt their enzyme content in response to developmental, metabolic and environmental cues via the import of new proteins rather than *de novo* synthesis of new organelles (Hayashi et al., 2000; Kaur and Hu, 2009; Palma et al., 2009). A defining feature of all peroxisome types (for which they are named), is their participation in the production and degradation of hydrogen peroxide through flavin-linked oxidases and catalase (CAT) respectively (Pracharoenwattana and Smith, 2008). Peroxisomes generate ROS, but can also rescue cells from the damaging effects of ROS. Accordingly, peroxisomes are the main site for renewal of cellular antioxidants and house an arsenal of antioxidant enzymes in addition to CAT, including superoxide dismutase, ascorbate peroxidase (APX), glutathione, and thioredoxin reductases (Lopez-Huertas et al., 2000; Corpas et al., 2001; Eubel et al., 2008).

For the nucleus, the initiation of a cell division cycle coordinates the ordered interaction of duplicated chromosomes with a microtubule (MT)-based spindle, ensuring that the nuclear genome partitions with stringent equality to each daughter cell (Franklin and Cande, 1999). For extranuclear organelles such as chloroplasts, mitochondria and the ER, there also appear to be specific, cytoskeleton-dependent mechanisms that ensure their unbiased inheritance during cell division (Sheahan et al., 2004, 2007b). However, little is known about processes that might act to ensure unbiased peroxisome inheritance in protoplasts initiating plant regeneration.

In *Arabidopsis* dividing suspension-cultured cells, peroxisomes replicate and segregate into daughter cells (Lingard et al., 2008). In dividing cells of onion roots peroxisomes redistribute into a ring circumscribing the inner edge of the expanding phragmoplast (Collings et al., 2003). However, the fact that all peroxisomal proteins are encoded by the nucleus and subsequently imported into the organelle suggests that peroxisomes may not need an active inheritance process (Subramani, 1993). Indeed, temperature sensitive yeast mutants that lack peroxisomes at the restrictive temperature can synthesize peroxisomes *de novo* if cells are placed at the permissive temperature (Waterham et al., 1993). However, there is no direct evidence for *de novo* synthesis in plants.

The induction of cell division in cultured plant protoplasts is associated with, and appears to require, an oxidative burst (Pasternak et al., 2002; Féher et al., 2008; Wang et al., 2011; Petřivalský et al., 2012). Thus, the dynamics of peroxisomes in cultured protoplasts may also reflect a response to excessive ROS. Excessive ROS can cause recalcitrance to regeneration, and processes to restore ROS homeostasis are required for efficient cell division (Cutler et al., 1991; Papadakis and Roubelakis-Angelakis, 1999; Papadakis et al., 2001; Petřivalský et al., 2012).

Using confocal microscopy to monitor fluorescently labeled peroxisomes during culture of *Arabidopsis* (and *Nicotiana*) protoplasts, we documented a massive amplification of the peroxisome population. By using cytoskeletal inhibitors, we show that the uniform redistribution of peroxisomes primarily depends on the actin and not MT cytoskeleton and that perturbing peroxisome dispersion greatly increased the bias in peroxisome inheritance. ROS levels declined rapidly during early protoplast culture in a manner inversely correlated with the increase in peroxisome number. The expression of peroxisome-related transcripts involved in the ascorbate-glutathione cycle remained essentially stable and inhibiting glutathione biosynthesis had little effect on ROS levels. Expression of the CAT isoform 3 (*CAT3*) and total CAT activity, however, increased dramatically during protoplast culture. Inhibiting CAT and catalase 3 mutants (*cat3*) resulted in increased ROS levels in cultured protoplasts. Moreover, *pex11a* and *cat3* mutants displayed a reduced induction of cell division compared to WT protoplasts cultures. The peroxisome dynamics may serve the functions of ensuring peroxisome maintenance and inheritance and facilitating an optimal redox subcellular environment for initiating totipotency.

## Materials and Methods

### Constructs and Plant Material

*Arabidopsis* (*Arabidopsis thaliana* ecotype Col-0) plants expressing a GFP fusion to peroxisomal multifunctional protein 2 (At3g06860; Cutler et al., 2000) were obtained from the ABRC (*Arabidopsis* Biological Resource Centre; CS84735). *Arabidopsis* plants expressing a mitochondria-localized mGFP5-ATPase fusion protein (Logan and Leaver, 2000) were the gift of Prof. David Logan. Homozygous *Arabidopsis pex11a* and *cat3* mutants were obtained from the ABRC with the stock number of SALK_038574C and CS327473 respectively. Kikume cDNA was amplified from the phKikGR1-S1 plasmid (Amalgam, Japan) using the primers 5′-CGGACGGGTCCGACCGGTTCAGCTTCGACATTCGCCGGCGGCGCGC-3′ and 5′-ATTTGCGGCCGCTTACAGCTTCGACTTGGCCAGCCTGGGCAGG-3′ which introduced an SKL (PTS1; peroxisome targeting signal 1) encoding sequence at the 3′ end of the cDNA and also SalI and NotI sites at the 5′ and 3′ ends, respectively. The resulting hKikGR1-SKL amplicon was cut by SalI/NotI and ligated into pENTR1A entry vector (Invitrogen, Carlsbad, CA, USA) before being recombined with the pMDC32 destination vector (Curtis and Grossniklaus, 2003) using LR clonase II enzyme (Invitrogen). The resulting pMDC32-hKikGR1-SKL construct was transferred into *Agrobacterium tumefaciens* (strain LBA4404) by electroporation and bacteria selected on LB agar with 50 μg⋅mL^-1^ kanamycin selection. Tobacco (*Nicotiana tabacum* cv. Xanthi) was stably transformed using the leaf disk procedure of (Horsch et al., 1985) as described in Sheahan et al. (2004). Tobacco plants expressing a mitochondria-localized coxIV-GFP fusion protein (Köhler et al., 1997) were the gift of Prof Maureen Hanson. Data presented are for *Arabidopsis* unless otherwise indicated.

### Plant Growth Conditions

*Arabidopsis* seedlings were grown horizontally in plates containing 0.5x Murashige and Skoog salts, 0.8% (w/v) agar and 1% (w/v) sucrose (Murashige and Skoog, 1962). Surface-sterilized seeds were positioned on plates and stratified for 2 days at 4°C before being moved to a controlled growth environment (22/18°C, 16/8 h photoperiod at 80 μmol photons m^-2^⋅s^-1^). Axenic cultures of tobacco shoots were established as described by Potrykus and Shillito (1986) and maintained in culture pots containing 1x MS medium, 0.8% (w/v) agar and 1% (w/v) sucrose in a controlled growth environment (25°C, 16/8 h photoperiod at 50 μmol photons m^-2^s^-1^).

### Protoplast Isolation and Culture

*Arabidopsis* mesophyll protoplasts were isolated from the aerial portion of 9 to 11-days-old plants as described in Sheahan et al. (2005) and were cultured at high density (∼2 × 10^5^ cells⋅mL^-1^) in KM8p culture medium (Kao and Michayluk, 1975). Tobacco mesophyll protoplasts were isolated from the leaves of axenic cultures, 3 weeks after subculture, and cultured in a modified NT medium with a light intensity of 0.5 μmol photons m^-2⋅^s^-1^ (Thomas and Rose, 1983) as described in Sheahan et al. (2004). In freshly isolated protoplasts cortical and perinuclear arrays remain largely intact and on incubation the density of AFs increase and transvacuolar arrays become prominent (Sheahan et al., 2004). MT immunofluorescence investigations with *Vicia hajastana* protoplasts show that while there is an initial disruption of MTs they re-organise and contribute to cell division in the normal way (Simmonds et al., 1983).

### Inhibitor Treatments

All inhibitors were obtained from Sigma (Sydney, NSW, Australia), except latrunculin B (Merck, Sydney, NSW, Australia) and oryzalin (Crescent Chemical Co., Singapore) and prepared as 1000 X stock solutions in dimethyl sulfoxide (final concentrations: latrunculin B; 1 μM, oryzalin; 10 μM, diphenyleneiodonium chloride; 25 μM, allopurinol; 200 μM, stigmatellin; 10 μM, myxothiazol; 10 μM and carbonyl cyanide 3-chlorophenylhydrazone; 0.5 μM) or water (final concentrations: 2-deoxyglucose; 2 mM, 3-amino-1,2,4-triazole; 10 mM). Protoplast cultures were exposed to inhibitors throughout the culture period for cytoskeletal inhibitors (latrunculin B, oryzalin) or for a 24 h period before analysis for inhibitors of the antioxidant machinery or ROS levels. Dimethyl sulfoxide [0.1% (v/v)] or water was used as a control in all experiments. While there is a small effect on cell volume with LatB and Oryzalin as cells progress toward division, cytokinesis is inhibited by LatB (40%) and by Oryzalin (80%).

### RNA Extraction, cDNA Synthesis and qPCR

Total RNA for each time point was isolated from protoplasts (3 mL) using an RNAqueous-micro kit (Ambion, USA) and DNase treated. Synthesis of cDNA was performed with 1 μg of total RNA, primed by oligo(dT), using a SuperScript III kit (Invitrogen). The cDNA was diluted 1:45 for quantitative real-time PCR (qPCR) reactions. All qPCR reactions were prepared using a CAS1200 robot (Qiagen) and run on a Rotor-Gene Q (Qiagen). Primers were designed using primer-BLAST^1^ such that at least one primer crossed an exon-exon boundary and that they produced amplicons of between 150 and 300 bp (Supplementary Table S1). Reactions were performed in duplicate using a qPCR mixture containing 0.3 U Platinum Taq and 1.5 μM SYTO9 fluorescent dye (Invitrogen). The qPCR cycling conditions were 94°C for 2 min, followed by 40 cycles of 94°C for 15 s, 57°C for 30 s and 72°C for 20 s. A dissociation curve was generated at the end of every run to ensure product uniformity. Gene expression was normalized to expression of polyubiquitin 10 (At4g05320) and relative expression calculated using the Pfaﬄ method (Pfaﬄ, 2001) with PCR efficiencies determined by LinRegPCR (http://linregpcr.nl/; Ramakers et al., 2003). Results shown are means ± SEM of at least three biological repeats and two technical repeats for each time point.

### Microscopy and Organelle Visualization

Protoplasts were mounted in welled slides and imaged using a LSM510 confocal laser- scanning microscope (Zeiss, Jena, Germany) equipped with a 40X C-Apochromat water-immersion objective. For RSR (Redox Sensor Red) staining, cells were incubated with the stain (1 μM) for 6 h before visualization. Green fluorescent protein or the green form of Kikume was visualized using the 488 nm Ar laser and BP500–530 filter, chloroplasts with the 543 nm He–Ne laser and a LP650 filter and RSR with the 543 nm He–Ne laser and BP565–615 filter. Images of protoplasts were acquired as z-stacks with a 1 μm interval as in previous studies on mitochondria (Sheahan et al., 2004). At least four protoplast preparations derived from independent plants or plates of seedlings were used to obtain quantitative data, with at least 15 cells imaged in each replicate.

### Image Analysis

To analyze peroxisome morphology we used 8-bit, greyscale z-stacks of half cells. Images were analyzed in ImageJ 1.47^2^ as described in Sheahan et al. (2004). Briefly, image scale was calibrated, the protoplast periphery was outlined (polygon selection tool) before limiting the threshold gray values between 25 and 254 and applying a binary threshold. The analyze particles function of ImageJ was used to assess peroxisome area, perimeter and number (particles with an area less than 2 pixels were excluded). For clustered or aggregated peroxisomes, the set threshold function was optimized at best to separate the individual peroxisomes. Clustering was defined as where three or more peroxisomes had touching faces in at least two optical sections, whereas cells with gross aggregation were defined as those having a large proportion of the peroxisome population sequestered in a localized region of cytoplasm. To assess peroxisome segregation outcomes, we compared the proportion of total peroxisomes, counted from serial optical sections (stacks), in each daughter cell half, using the ‘analyze particles’ function of ImageJ. Peroxisome inheritance was also assessed by comparing the volume of peroxisomes (calculated as the summed plan-area) in each daughter cell half. Plan area is calculated as the sum of the surface areas of each optical section, using at least 100 cells from three biological replicates. The proportion of mitochondrial GFP and RSR colocalisation was determined by overlaying the red (RSR) and green (mtGFP) channels in Photoshop CS5 (Adobe Systems, San Jose, CA, USA), with yellow (overlay) relative to the proportion of total RSR stain in a cell, using at least 12 cells from three biological replicates.

### Analysis of Ascorbate and Glutathione

Approximately 20 mg of protoplasts were collected by centrifugation, the supernatant removed and the cells immediately frozen in liquid nitrogen, before determining ascorbate and glutathione levels as previously described (Law et al., 1983; Griffith, 1985; Smith, 1985; Zhang and Kirkham, 1996). Samples were assayed at 24 h intervals, with at least three biological and two technical repeats examined for each time point.

### Measurement of Enzyme Activity

Protoplasts (600 μL) were collected by centrifugation, the supernatant removed and immediately frozen in liquid nitrogen. The activity of CAT, APX, and glutathione reductase (GR) was assayed essentially as described (Maruthasalam et al., 2010). Briefly, for assessment of CAT activity, the protoplast pellet was resuspended in 500 μL CAT assay buffer (50 mM phosphate buffer pH 7.0, 0.1 μM EDTA) and the cells disrupted by pulse sonication (Hielscher sonicator UP50H; 3 × 15 s at 80% amplitude and 0.5 s cycle). Disrupted cells were allowed to cool on ice before proceeding. Measurement was initiated by the addition of H_2_O_2_ (40 mM final concentration) and the decrease in absorbance of H_2_O_2_ monitored at 240 nm with a spectrophotometer (Thermo Spectronic BioMate 3) for 60 s, with readings taken every 10 s. Total CAT activity was calculated as μmol of H_2_O_2_ decomposed min^-1^⋅mg^-1^ of fresh weight. To analyze APX activity, protoplasts were resuspended in a 500 μL APX assay buffer (50 mM phosphate buffer pH 6.0, 0.1 μM EDTA, 0.5 mM ascorbate) and sonicated. Measurement was initiated by the addition of H_2_O_2_ (20 mM final concentration) and the decrease in absorbance of ascorbate at 290 nm was monitored for 60 s, as described above, and APX activity calculated as μmol of ascorbate oxidized min^-1^⋅mg^-1^ of fresh weight. To analyze GR activity protoplasts were resuspended in 500 μL of GR assay buffer (50 mM phosphate buffer pH 7.8, 0.1 μM EDTA, 0.05 mM NADPH) and sonicated. Measurement was initiated by the addition of GSSG (3 mM final concentration) and the decrease in absorbance of NADPH at 340 nm was monitored for 60 s, as described above, and GR activity calculated as μmol NADPH oxidized min^-1^⋅mg^-1^ of fresh weight. Measurements for enzyme activity were obtained from at least three biological replicates.

### Measurement of Reactive Oxygen Species Level

Extracellular H_2_O_2_ production was measured in control, inhibitor-treated and mutant protoplast populations using a modification of the luminol-based chemiluminescence assay described by Murphy and Huerta (1990). At each time-point, 3 mL of protoplast suspension was collected by centrifugation (100 *g*, 10 min) and the resulting pellet resuspended in 1 mL of luminometry assay buffer (0.6 M mannitol, 10 mM MES pH 7.0, 1 mM CaCl_2_, 0.1 mM KCl). Luminescence measurements were made on a luminometer (Berthold Lumat 953, Germany). To each tube, 5 μL of 25 mM luminol and 5 μL of 10 U⋅μL^-1^ horseradish peroxidase (type VI-A; Sigma) was added, the tube shaken briefly (<1 s) and luminol-dependent luminescence measured immediately. The assay time was 120 s with nine measurements taken for 5 s each. A buffer-only control (luminometry assay buffer, luminol and horseradish peroxidase) was run in parallel for every measurement. The average of the final seven values for the buffer control was subtracted from the average of the final seven values from the cells to obtain the final reading. To examine H_2_O_2_ production in macerated leaf tissues, a leaf disk, equivalent in fresh weight to the calculated mass of protoplasts (∼50 mg) in the luminometry reaction, was sliced into 2 mm strips, immediately placed in luminometry buffer with pre-added luminol and horseradish peroxidase and the chemiluminescence measured. Alternatively, measurements for ROS levels (**Figures 4B,D**) spectrophotometry was used as described by Maruthasalam et al. (2010), with three biological replicates.

## Results

### Mesophyll Protoplast Culture and Peroxisomes in Isolated *Arabidopsis* Protoplasts

**FIGURE 1:**
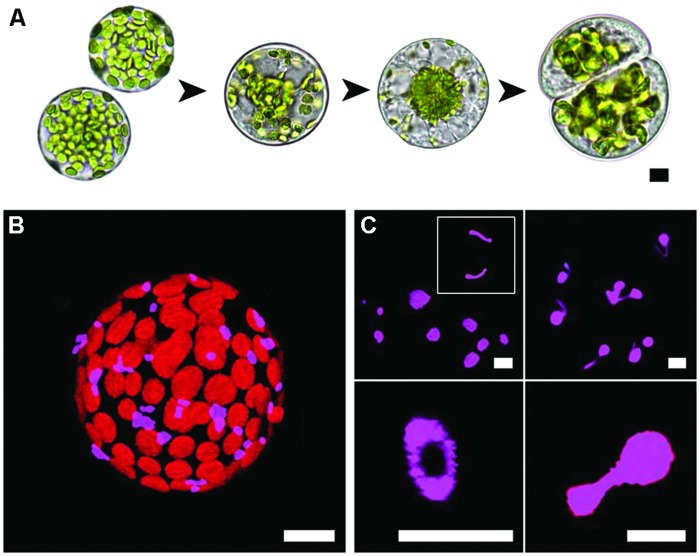
**Mesophyll protoplast culture and peroxisomes in isolated protoplasts. (A)** The experimental system used in this study. Isolated protoplasts dedifferentiate, the cell wall is renewed and cell division follows. Note how the chloroplasts move to the nucleus then partition in similar numbers to the daughter cells. **(B)** Sparse distribution and co-localisation of peroxisomes with chloroplasts in a freshly isolated protoplast. **(C)** Confocal images of different peroxisome morphologies observed during early (<24 h) protoplast culture, clockwise from top left; spherical and variably sized peroxisomes or elongated peroxisome forms (inset), threadlike extensions emanating from spherical peroxisomes, toroidal forms and dumbbell-shaped peroxisomes. Bars = 10 μm **(A,B)** or 5 μm **(C)**.

*Arabidopsis* mesophyll protoplasts dedifferentiate and undergo cell division during culture (**Figure 1A**). Peroxisomes in freshly isolated protoplasts are sparsely distributed and are in close association with the chloroplasts (**Figure 1B**). Though generally spherical (**Figure 1C**, top left), as is common for peroxisomes, there can be a diversity of shapes. Some elongated forms are evident (**Figure 1C**, top left; inset), a number of cells exhibited peroxisomes with fine and highly mobile threadlike extensions (**Figure 1C**, top right). Although less common, toroidal (**Figure 1C**, bottom left) and dumbbell forms (**Figure 1C**, bottom right) were also observed, with dumbbells possibly representing peroxisome-division intermediates. The average peroxisome area in the freshly isolated protoplasts was 2.2 ± 0.11 μm^2^.

### Peroxisome Proliferate During Protoplast Culture

**FIGURE 2:**
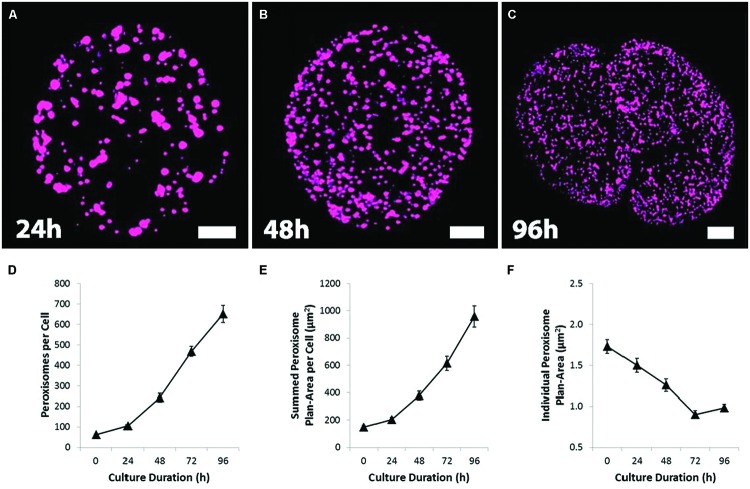
**Proliferation, dispersion and partitioning of the peroxisomes during protoplast culture and division. (A–C)** Confocal projection images showing the change from a large, sparsely distributed peroxisome population to a small, numerous and uniformly dispersed population during protoplast culture, followed by protoplast division. Bars = 10 μm. **(D)** Quantification of the number of peroxisomes showing the dramatic increase in peroxisome number during protoplast culture. **(E)** The total volume of peroxisomes per cell, gaged by integrating peroxisome plan-areas. **(F)** Quantification of peroxisome plan-area. Values are means ± SEM with *n* ≥ 100 **(D–F)**.

In fresh protoplast preparations, there were on average, 62 ± 5 (mean ± SEM, *n* ≥ 100) peroxisomes per cell. The number of peroxisomes increased dramatically over protoplast culture, however, increasing more than 10-fold by 96 h of culture (**Figure 2D**). Given their plan area (**Figures 2A,F**; Supplementary Figures S5A,C), some peroxisomes in early culture could have a shape and orientation that would give a vertical length greater than 1 μm and be counted twice. This would mean the starting number is slightly lower and making the 96 h fold increase slightly higher.

The total volume of peroxisomes per cell, as gaged by peroxisome plan-area (sum of peroxisome surface area from each 1 μm section of a cell), also increased dramatically (**Figure 2E**); even though individual peroxisomal plan-area decreased during culture (**Figure 2F**). These changes increase the surface area of the peroxisome population and as such may facilitate a more efficient movement of molecules across the peroxisome membrane. The increased peroxisome numbers and surface areas is associated with increased expression of genes associated with the division and biosynthesis of peroxisomes (Supplementary Figure S1). As the peroxisomes increased in number, the changes to peroxisome distribution and morphology closely mirror those of mitochondria (Sheahan et al., 2005). Initially sparsely distributed, relatively large and clustered around chloroplasts (**Figure 1B**), peroxisomes dispersed near uniformly throughout the cytoplasm before the first cell division (**Figures 2A–C**).

### Peroxisome Inheritance During Protoplast Culture

**FIGURE 3:**
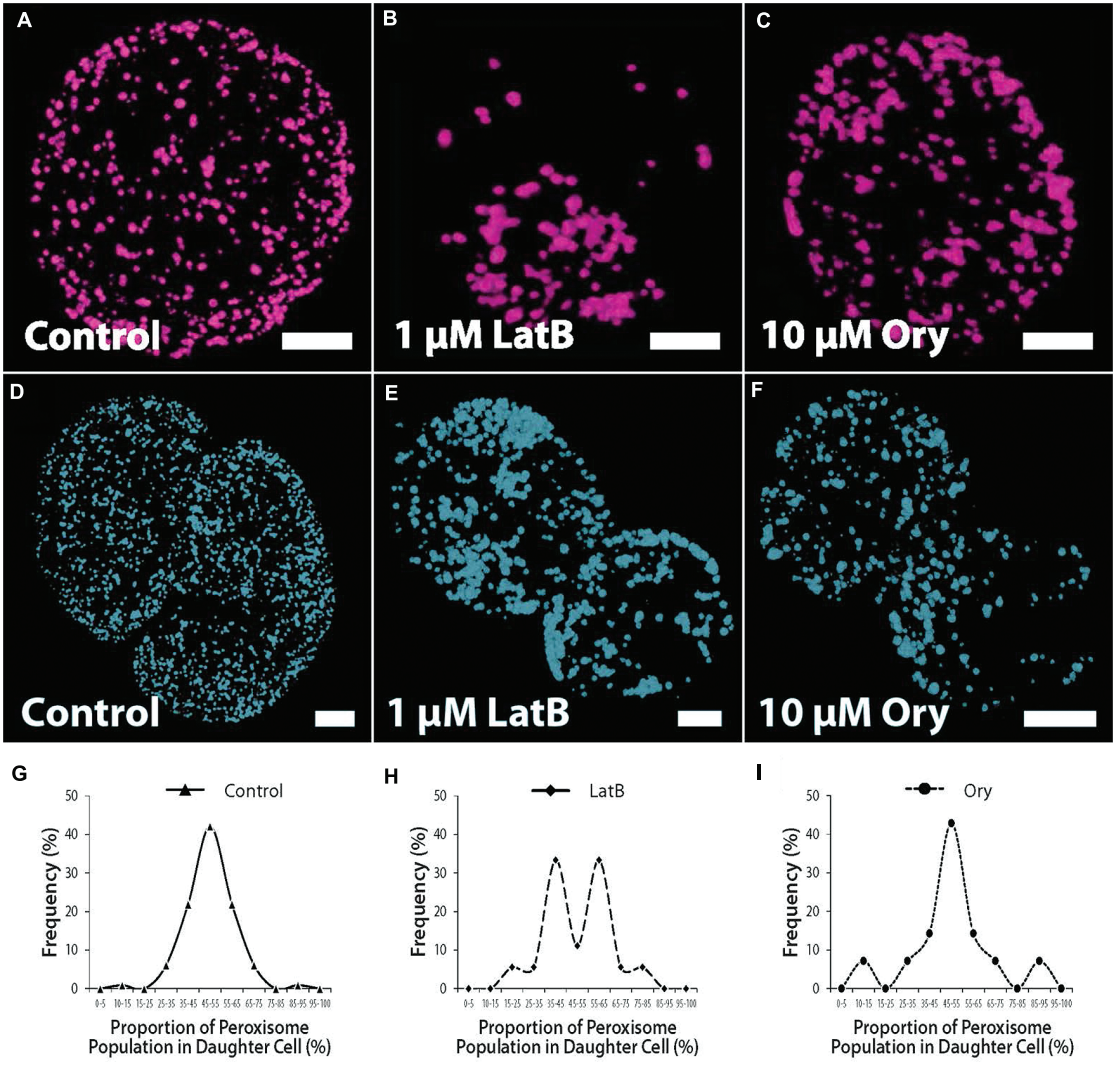
**Peroxisome dispersion, inheritance and cytoskeletal inhibitor studies. (A–C)** Confocal projection images showing peroxisome distribution in control and inhibitor-treated protoplasts after 72 h culture. **(A)** Uniformly distributed peroxisomes in controls. **(B)** Clustered and grossly aggregated peroxisomes in LatB-treated cells. **(C)** Clustering of peroxisomes, but with fewer tendencies for gross aggregation relative to LatB-treated cells in Ory-treated cell cultures. **(D–F)** Peroxisome inheritance in dividing protoplasts. **(D)** Peroxisome distribution in controls. **(E)** Peroxisome distribution after LatB-treatment. **(F)** Peroxisome distribution after Ory-treatment. **(G)** Controls with proportion of peroxisomes in daughter cells showing small deviation from equal inheritance. **(H)** LatB-treated and **(I)** Ory-treated protoplasts showing biased inheritance of protoplasts. Data are from symmetrically divided protoplasts. Bars = 10 μm. Values are means ± SEM with *n* ≥ 100.

We surmised that the uniform dispersion of numerous peroxisomes might represent an inheritance strategy, similar to that of mitochondria (Sheahan et al., 2004). We therefore compared peroxisome inheritance in control and cytoskeleton-disrupted cells. In cells treated with the actin filament (AF)-disrupting agent, Latrunculin B (LatB; 1 μM), peroxisomes were less uniformly distributed compared to controls (**Figures 3A,B**), typically concentrating in localized regions of the cell and often co-localizing with chloroplasts. Peroxisomes in LatB-treated cells also formed larger aggregate complexes (**Figure 3B**). In cells treated with the MT-disrupting agent, oryzalin (Ory; 10 μM), peroxisome clustering and aggregation also appeared more prevalent relative to controls (**Figure 3C**), but not to the extent of LatB treatment. A random sampling of cells revealed that LatB-treated cells (*n* = 73) resulted in almost 1.5-fold more cells exhibiting clustered peroxisomes relative to controls (*n* = 89), whereas Ory-treated cells (*n* = 92) caused a more modest, 1.2-fold increase (Supplementary Figure S2). More importantly, LatB and Ory treatment increased the proportion of cells with grossly aggregated peroxisomes by 3.5- and 1.9-fold relative to controls (Supplementary Figure S2). The number of peroxisomes in daughter cell pairs in symmetrically divided cells of control cultures, were shown to be uniformly distributed between daughter cells (**Figure 3D**). Accordingly, there was a small (6.6%) deviation from equal inheritance (SD, *n* = 88 cells; **Figure 3G**). The reduced number and less uniform distribution of peroxisomes in LatB- or Ory-treated protoplasts clearly affected inheritance outcomes (**Figures 3E,F**). In LatB- and Ory-treated protoplasts, the deviation from equal segregation was 21% (SD, *n* = 72 cells) and 20% respectively, a 3-fold increase in the segregation bias relative to controls. Most cell divisions in cytoskeleton-disrupted protoplasts were, however, asymmetric. In controls, 68% of divisions were symmetric, whereas the respective values for LatB- and Ory-treated protoplasts were 27 and 19% (data not shown). An analysis of only symmetrically divided cells revealed a similar tendency for biased peroxisome segregation in LatB-treated cultures, with a bimodal distribution of inheritance frequencies (**Figure 3H**). In contrast, segregation bias in Ory-treated cultures was substantially reduced (**Figure 3I**). Analysis of peroxisome inheritance by peroxisome volume also generally reflected the results derived from peroxisome number.

### ROS Production Associated with Protoplast Isolation and Culture

**FIGURE 4:**
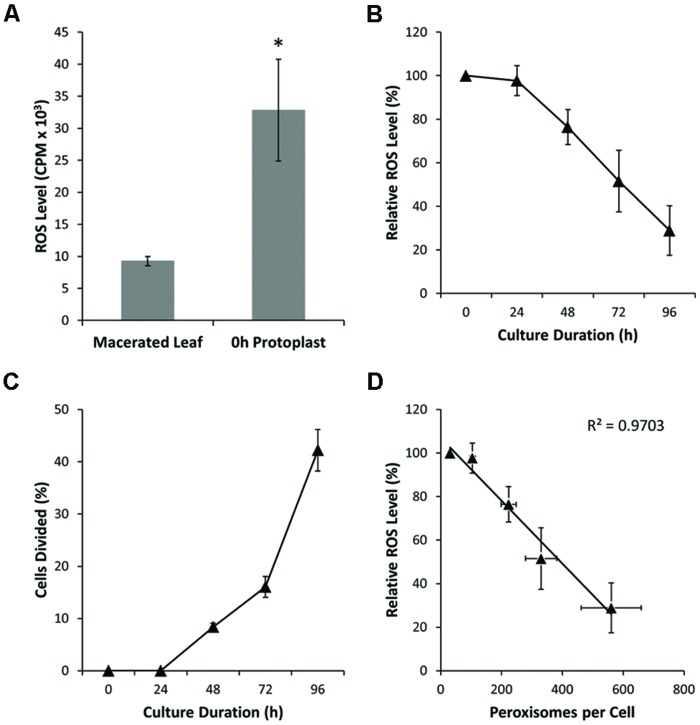
**Peroxisome proliferation and ROS detoxification. (A)** Hydrogen peroxide production as measured in macerated leaves (source tissue for protoplast isolation) and in freshly prepared (0 h) protoplasts. Note substantial increase in ROS level for fresh protoplasts. **(B)** Hydrogen peroxide levels in cultured protoplasts decrease during culture and before cellular division. **(C)** Cell division rate for cultured protoplasts. Cell division occurs after 48 h culture. **(D)** Correlation between peroxisome number and ROS level (*R*^2^ = 0.97). Values are means ± SEM with *n* ≥ 3 (^∗^*p* < 0.05, *t*-test).

**FIGURE 5:**
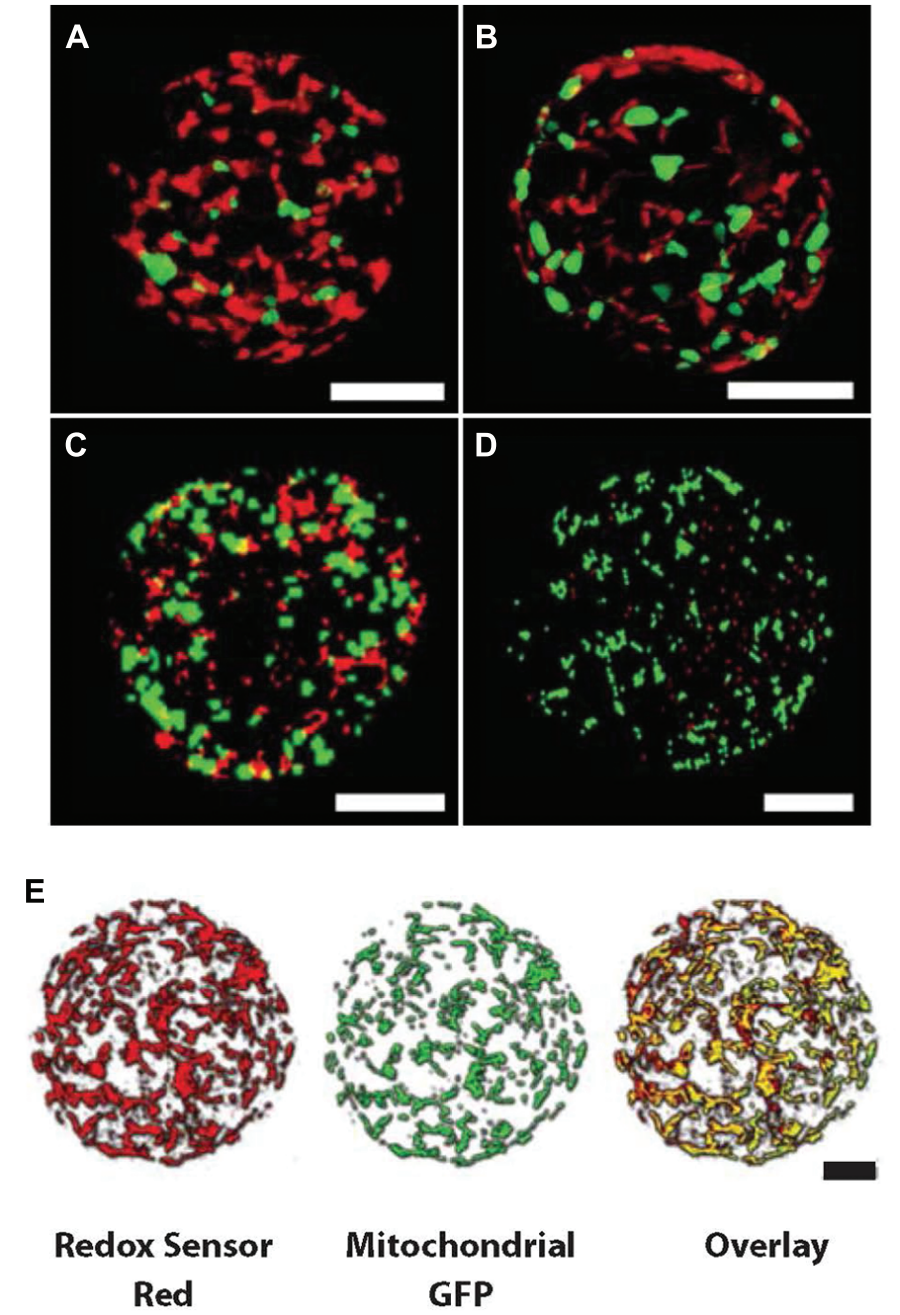
**Mitochondria expressing GFP labeled with Redox Sensor Red (RSR) show a decrease in number as ROS levels decrease during protoplast culture. (A–D)** Confocal projection images of *Nicotiana* protoplasts incubated for 6 h in RSR, clockwise from top left; 24, 48, 72, and 96 h. The number of RSR (red) stained mitochondria become fewer as ROS levels decrease (also see Supplementary Figure S4). Green represents GFP-labeled mitochondria with no RSR staining. Bars = 20 μm **(A–D)**. **(E)** Overlay of confocal images of both RSR and GFP (yellow) at the beginning of incubation showing most subcellar ROS arise from the mitochondria. Bars = 10 μm **(E)**.

Stress-induced ROS has been suggested as an important inductive signal in plant cells acquiring totipotency (Pasternak et al., 2002; Féher et al., 2008; Faltin et al., 2010; Wang et al., 2011). Therefore, using chemiluminescence assays, we assessed extracellular H_2_O_2_ production in cultured mesophyll protoplasts. These assays revealed an elevated ROS production in freshly isolated protoplasts relative to macerated leaves (**Figure 4A**; Supplementary Figure S3A). During protoplast culture, however, measured levels of extracellular H_2_O_2_ decreased substantially, notably before significant rates of cell division were observed (**Figures 4B,C**; Supplementary Figures S3B,C). By utilizing RSR (a probe that labels subcellular ROS) to stain protoplast cell cultures, we confirmed the findings of the chemiluminescence analysis, with the proportion of RSR-stained mitochondria decreasing during protoplast culture (**Figures 5A–D**; Supplementary Figure S4). There was a strong, negative correlation between ROS level and peroxisome number in protoplasts (**Figure 4D**; Supplementary Figure S3D). These results indicate that ROS levels are perturbed by protoplast isolation, but then ROS homeostasis is largely restored before cell division and concomitant with an increase in peroxisome number.

### Source of ROS and Mechanisms of Homeostasis

To further investigate the nature of the homeostatic mechanisms in operation to regulate ROS levels in protoplasts, we investigated the source of ROS production by applying agents that inhibit ROS production from different cellular sources (**Table 1**). As expected application of DPI inhibited ROS production almost entirely, given that DPI inhibits flavoproteins particularly NADPH oxidase and mitochondria NADH-ubiquinone oxidoreductase (Li and Trush, 1998; Riganti et al., 2004). Inhibition of glycolysis by 2-DOG also resulted in decreased ROS production, whereas inhibition of xanthine dehydrogenase-oxidase, a cytosolic enzyme that produces H_2_O_2_, had little impact on ROS levels. Surprisingly, however, inhibitors of mitochondrial electron transport also dramatically reduced ROS production, as did the uncoupling agent CCCP. In the case of stigmatellin the contribution of chloroplasts would be minimal as the light intensity is very low and chloroplasts are dedifferentiating toward proplastids (Thomas and Rose, 1983). Overall these findings suggest that mitochondria are the major contributors to the extracellular H_2_O_2_ measured in protoplast cultures. Consistent with these data are the overlayed confocal images of RSR stained protoplast cultures with GFP-labeled mitochondria (**Figure 5E**) at the beginning of the incubation period, when mitochondria stain with RSR as a result of ROS production and before the mitochondria become reduced as ROS is detoxified.

**Table 1:** Source of reactive oxygen species (ROS) production in protoplasts as indicated by inhibitors.

Inhibitor (Abbreviation)	Concentration	Mechanism of action	ROS Production (%)^a^
Control (DMSO)	–	Vehicle for inhibitors	100 ± 1.1
Diphenyleneiodonium chloride (DPI)	25 μM	General inhibitor of flavoproteins (e.g., NADPH oxidases, mitochondrial complex I)	22.8 ± 14.4
2-Deoxyglucose (2-DOG)	2 mM	Competitive inhibitor of hexokinase. Restricts flux of metabolites through glycolysis and decreases production of reductants (NADH)	63.4 ± 2.0
Allopurinol (AP)	200 μM	Inhibitor of Xanthine Dehydrogenase-Oxidase	95.5 ± 10.3
Stigmatellin (Stig)	10 μM	Binds Q_0_ site of mitochondrial complex III and prevents oxidation of ubiqinone, stopping mitochondrial electron transport (in the absence of AOX)	29.9 ± 1.3
Myxothiazol (Myx)	10 μM		34.8 ± 2.0
Carbonyl cyanide 3-chlorophenylhydrazone (CCCP)	0.5 μM	Protonophore. Uncouples mitochondrial electron transport from oxidative phosphorylation	18.9 ± 4.3

*^a^Relative to control cultures. Mean ± SEM, *n* = 3.*

### What Drives the Reduction in ROS Levels before the Onset of Cell Division?

**FIGURE 6:**
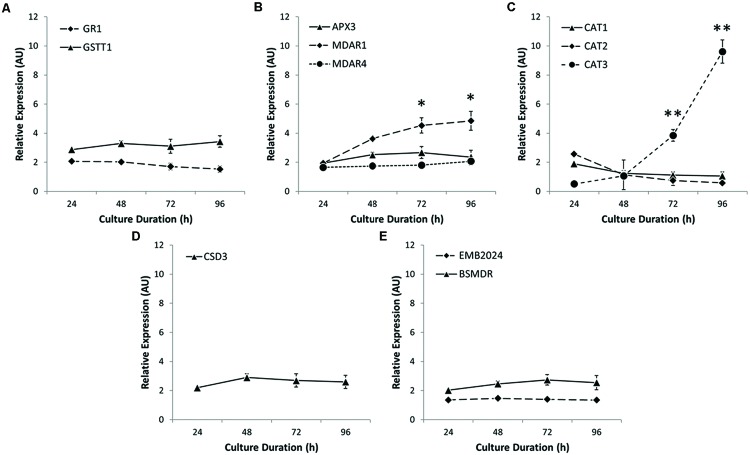
**Expression profiling of peroxisome-localized antioxidants. (A)** Glutathione *S*-transferase 1 (*GSTT1*) and glutathione reductase 1 (*GR1*) show minimal increases in expression during protoplast culture. **(B)** Monodehydroascorbate reductase 1 (*MDAR1*) is upregulated during protoplast culture, whereas peroxisomal ascorbate peroxidase 3 (*APX3*) and *MDAR4* show little change. **(C)** Only *CAT3* showed a significant increase in expression. **(D)** Copper Superoxide Dismutase 3 (*CSD3*) showed continuous unchanged expression levels. **(E)** Other antioxidant-related enzymes such as bifunctional short- and medium-chain dehydrogenase/reductase (*BSMDR*) and a 6-phosphoglucunolactonase encoded by a gene product embryo defective 2024 (*EMB2024*) also showed little change in expression during culture. Values are means ± SEM with *n* ≥ 3. (^∗^*p* < 0.05, ^∗∗^*p* < 0.01, *t*-tests).

Since cellular ROS levels represent a balance between ROS generation and degradation, the reduction in ROS level observed may result from decreased ROS generation or increased antioxidant capacity. Enzymatic antioxidants that catalyze the breakdown of H_2_O_2_ include enzymes such as APX and CAT. We investigated the expression profile of key peroxisome-localized gene products involved in ROS metabolism (**Figure 6**). Peroxisome-localized glutathione-related enzymes, glutathione *S*-transferase 1 (*GSTT1*) and glutathione reductase (*GR1*) showed little change in steady-state transcript levels (**Figure 6A**), as did the monodehydroascorbate (MHA) reducing enzyme *MDAR4* and *APX3* (**Figure 6B**). The levels of *MDAR1* transcript did, however, increase significantly over time. The most striking change in expression profile, however, was the more than 10-fold up-regulation of the specific *CAT3* isoform of *CAT* after 96 h culture (**Figure 6C**), coincidently, when ROS levels drop most precipitously in *Arabidopsis* protoplasts (**Figure 4B**). Levels of a peroxisome-localized copper superoxide dismutase (*CSD3*) and two other transcripts which code for enzymes associated with other aspects of ROS metabolism were essentially unchanged (**Figures 6D,E**).

### Ascorbate and Glutathione are Abundant and Reduced in Cultured Protoplasts

**FIGURE 7:**
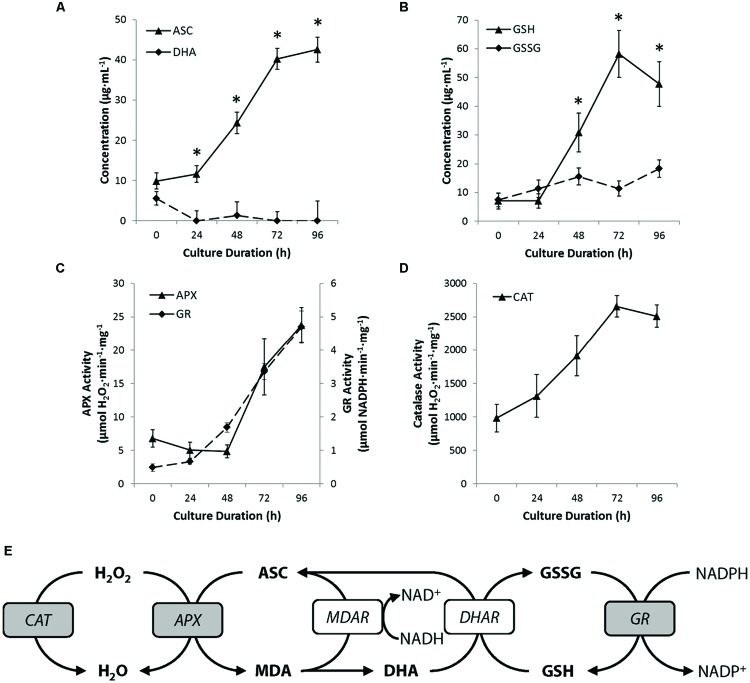
**Peroxisomes promote ROS homeostasis. (A,B)** Quantifying levels of reduced and oxidized ascorbate **(A)** or reduced and oxidized glutathione **(B)** revealed that both antioxidants are maintained in a reduced state and that the concentration of both molecules increased during culture. **(C)** Activity of ascorbate peroxidase (APX) and glutathione reductase (GR) in cultured protoplasts increased steadily after 48 h culture. **(D)** The activity of catalase increased through the early phase of culture. Note that the activity of catalase was at least 50-fold greater than that of maximum APX activity **(E)** Schematic summary of the key antioxidant cycles in peroxisomes. Enzymes assayed shown in gray. Abbreviation used are: APX, ascorbate peroxidase; ASC, ascorbate; CAT, catalase; DHA, dehydroascorbate; DHAR, dehydroascorbate reductase; GR, glutathione reductase; GSH, glutathione (reduced); GSSG, glutathione disulphide; MDA, monodehydroascorbate; MDAR, monodehydroascorbate reductase. NADH/NAD^+^, nicotinamide adenine dinucleotide (reduced/oxidized); NADPH/NADP^+^, nicotinamide adenine dinucleotide phosphate (reduced/oxidized). Values are means ± SEM with *n* ≥ 3. (^∗^*p* < 0.05, *t*-test).

To examine in more detail, the redox environment within cultured protoplasts, we profiled whole-cell ascorbate and glutathione levels and oxidation status. During early culture (0–24 h), when ROS levels are at their peak (**Figure 4B**), ascorbate and glutathione concentrations remained essentially unchanged (**Figures 7A,B**). In the next 48 h of culture, however, both ascorbate and glutathione concentrations increased approximately fourfold, after which time, changes in ascorbate and glutathione concentrations stabilized. Moreover, and intriguingly, both antioxidants were maintained in a predominantly reduced state after 24 h of culture initiation (**Figures 7A,B**). This surprising finding suggests that either the activity of reductive enzymes in the ascorbate-glutathione cycle (see **Figure 7E**) is maintained in excess of what is required, or alternatively, that ascorbate and glutathione are not acting as ‘frontline’ antioxidants in the restoration of ROS homeostasis in cultured protoplasts.

### Restoration Versus Maintenance: Differing Roles for Ascorbate/Glutathione and Catalase

We therefore examined the activity of two key enzymes of the ascorbate-glutathione cycle; APX and GR. The total cellular activity of APX, which oxidizes ascorbate to dehydroascorbate (see **Figure 7E**), was consistently low during the first 48 h of culture, but thereafter, increased linearly to approximately fourfold the activity of freshly isolated protoplasts by the end of culture (**Figure 7C**). Similarly, total cell activity for GR, an enzyme that reduces glutathione disulphide to glutathione (see **Figure 7E**), was low during early culture (0–24 h) and then increased linearly to approximately 6.5-fold initial activity by the end of protoplast culture (**Figure 7C**). The activity profile for GR correlates well with the levels of reduced glutathione (GSH) measured (**Figure 7B**) and suggests that the predominance of reduced glutathione may at least in part be attributable to the high activity of GR. However, assuming ROS production in protoplasts is constant or increases during culture, the profile for APX activity does not correlate well with measured ROS levels (compare **Figures 4B and 7C**), reinforcing the notion that ascorbate is not a ‘frontline’ antioxidant involved in the restoration of ROS homeostasis in cultured protoplasts.

Finally, we examined CAT, a peroxisome-specific enzyme that reduces H_2_O_2_ to water. CAT activity increased linearly by approximately 2.5-fold during the first 72 h of culture and then plateaued (**Figure 7D**). Interestingly, the theoretical H_2_O_2_-removal capacity conferred by CAT is at least 100-fold greater than that for APX, throughout culture. Moreover, the plateau in CAT activity (72–96 h) coincided with the plateau or decline in concentrations of ascorbate and glutathione, respectively (compare **Figures 7A,B,D**). Taken together, our data suggest that ascorbate and glutathione play a subservient role to CAT in the restoration of ROS homeostasis following the wound-induced oxidative burst in protoplasts, whereas ascorbate and glutathione may play an important role in the maintenance of ROS homeostasis, once it is attained.

### Catalase and the Restoration of ROS Homeostasis During Protoplast Culture is Required for Optimal Regeneration Capability During Protoplast Culture

**FIGURE 8:**
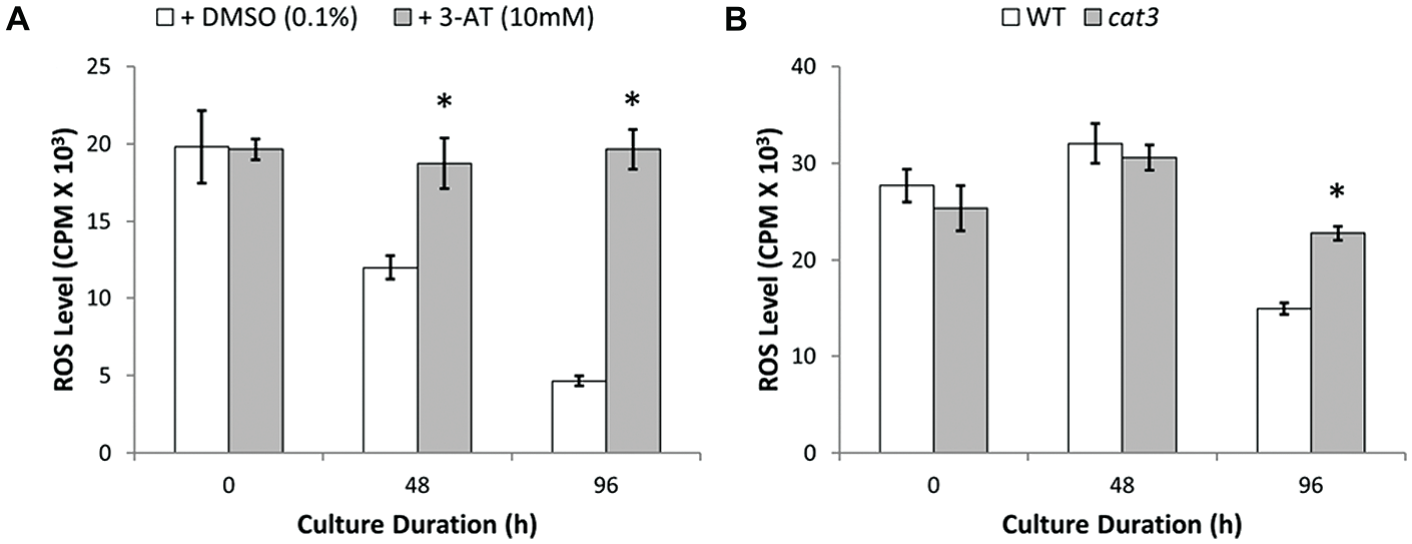
**Elevated ROS levels observed in catalase inhibited protoplasts with 3-AT and *cat3* mutant protoplast cultures. (A)** Increased ROS levels observed throughout culture in catalase inhibited protoplasts treated with 3-AT for 24 h. **(B)** Homozygous *cat3* mutant protoplasts significantly increased ROS levels after 96 h culture. Values are means ± SEM with *n* ≥ 3. (^∗^*p* < 0.05, *t*-test).

To confirm the importance of CAT to the restoration of ROS homeostasis, we treated protoplasts with the CAT inhibitor 3-amino-1,2,4-triazole (3-AT, 10 mM; (Margoliash and Novogrodsky, 1958; Jannat et al., 2011) for 24 h and examined H_2_O_2_ levels. Protoplast cells treated with 3-AT maintained ROS generation at a consistently high level throughout culture (**Figure 8A**). However, similar treatment of cells with buthionine sulfoximine (2 mM), an inhibitor of the γ-glutamylcysteine synthase enzyme required for glutathione biosynthesis, had no influence (3.6 ± 8.6%) on the measured levels of H_2_O_2_. Additional evidence for the importance of CAT comes from the *cat3* mutant, where increased ROS levels of approximately 50% (*n* ≥ 5) were observed in *cat3* mutant protoplasts compared to WT protoplasts after 96 h culture (**Figure 8B**).

**FIGURE 9:**
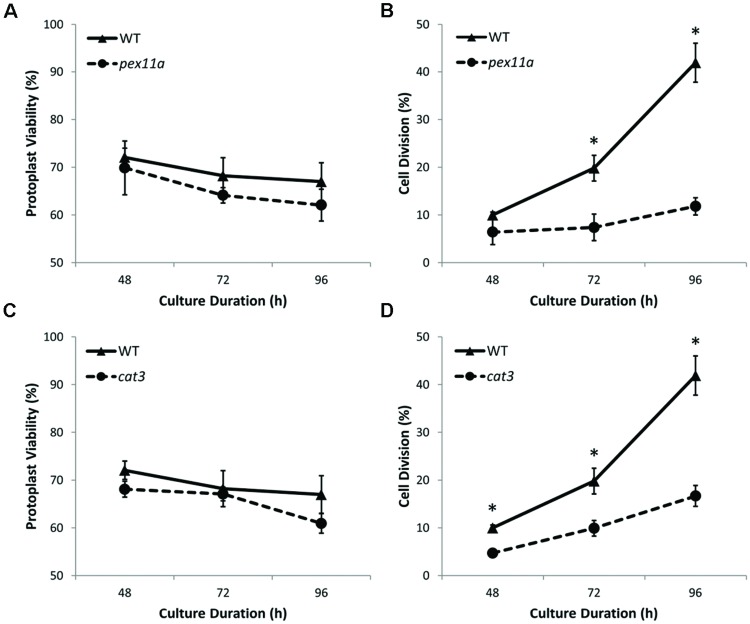
**Mutants of both *PEX11a* and *CAT3* protoplast cultures exhibited reduced cell division rates. (A,B)** Viability of *pex11a* and *cat3* protoplast cultures decreased by approximately 5% compared to WT cultures over 96 h. **(C,D)** A delayed cell division initiation with reduced cell division rates by approximately 3.5- and 2.5-fold for *pex11a* and *cat3* cultures respectively after 96 h. Values are means ± SEM with *n* ≥ 3. (^∗^*p* < 0.05, *t*-test).

To further investigate the influence of peroxisomes and redox balance on plant cell regeneration during protoplast culture, homozygous mutant protoplasts of *PEX11a* and *CAT3* were cultured in parallel with WT protoplasts. Viability of both *pex11a* and *cat3* protoplast cultures decreased by approximately 5% after 96 h culture (**Figures 9A,B**). However, after 96 h culture, reduced cell division rates of approximately 3.5- and 2.5-fold for *pex11a* and *cat3* protoplasts were observed (**Figures 9C,D**). These data are consistent with a requirement for peroxisomes and CAT and their modulation of redox homeostasis for the initiation of cell division in cultured protoplasts.

## Discussion

### Peroxisome Dynamics and AF-Dependent Inheritance in Cells Reprogrammed to Divide

The large proliferation of peroxisomes and their uniform distribution throughout the cell prior to cell division is the main contributor to their partitioning with minimal bias to daughter cells. These changes that promote unbiased inheritance suggest that it is advantageous to ensure the presence of peroxisomes in nascent daughter cells. This would still be the case even if plant protoplasts were capable of *de novo* peroxisome biogenesis, a possibility suggested by studies in yeast (Nagotu et al., 2010), but with no evidence in plants (Hu et al., 2012).

Disruption of AFs, and to a lesser extent MTs, perturbed the dispersion of peroxisomes, leading to their aggregation and increased segregation bias. In plant cells, peroxisome motility depends on both AFs and myosin (Mathur et al., 2002; Avisar et al., 2008) supporting the inability of peroxisomes to redistribute uniformly in the cytoplasm of LatB-treated protoplasts. MTs, however, also appeared to have a role, albeit minor, in peroxisome dispersion. In animal cells, where peroxisome motility is predominantly MT dependent, AFs seem to play a cooperative role in peroxisome motility (Rapp et al., 1996). Mitochondrial motility in plants is also AF-dependent, yet their positioning within the cell is regulated by both MTs and AFs (Van Gestel et al., 2002). Therefore potentially, MTs may play similar roles in peroxisome motility and positioning. The action of oryzalin on nucleus positioning and cytokinesis, in our protoplast system, is consistent with MT inhibition (Sheahan et al., 2004). However, while the oryzalin pharmacological observations point to a limited role of MTs in peroxisome dispersion, detailed studies of MT dynamics are required.

### Peroxisome Dynamics During Dedifferentiation and Division Induction Compared to other Organelles

The increase in peroxisome numbers, their uniform dispersion throughout the cytoplasm followed by unbiased inheritance to daughter cells is similar to what occurs with mitochondria (Sheahan et al., 2004). The mitochondria do undergo massive fusion (Sheahan et al., 2005) prior to fission, but the cytoskeletal dependent dispersal prior to division is similar to peroxisomes. Though AF dependent movement of peroxisomes is known (Wada and Suetsugu, 2004) the cytoskeleton inhibitor analysis indicates that it is the AFs that are primarily responsible for the uniform dispersion and this enables the unbiased partitioning. What occurs with the chloroplasts is, however, quite different. Chloroplasts, possibly because they are much larger than mitochondria and peroxisomes, are reproducibly translocated from the protoplast cortex to the perinuclear region (see Sheahan et al., 2004 with supplementary video) to achieve unbiased partitioning. The perinuclear clustering appears to be driven by a process involving a dynamic re-organization of the actin network followed by entrapment adjacent to the nucleus (Sheahan et al., 2004; **Figure 1A**). In differentiated cells such as the leaf mesophyll cells there are well established associations of peroxisomes and chloroplasts (Schumann et al., 2007; **Figure 1B**) and they distribute together (Wada and Suetsugu, 2004).

The chloroplasts do not divide as they dedifferentiate to proplastids before division; plastid division occurring from the 4 cell stage (Thomas and Rose, 1983). What is clear is that the pathway to totipotency is highly ordered with the cytoskeleton ensuring the unbiased inheritance of the organelles.

### Integrating Organelle Inheritance Processes with Cell Growth, the Cell Cycle and the Cytoskeleton

The mechanisms by which information about cell volume is conveyed to cell cycle regulators remains poorly understood (Jorgensen et al., 2002). Disruption of AFs or MTs does not appear to have a large effect on cell expansion in cultured protoplasts, however, numbers of mitochondria (Sheahan et al., 2004) and peroxisomes (Supplementary Figure S5) are consistently lower in cytoskeletal inhibitor-treated cells compared to controls. Other mechanisms, such as those involving redox homeostasis (Foyer and Noctor, 2005), independent of cell volume signaling, are also likely to influence peroxisome dynamics and cell cycle progression in protoplasts.

### Redox, Dedifferentiation and Cell Division Induction

A very rapid oxidative burst is an important stress signal in isolated plant cells or tissue which is likely part of the signaling, in conjunction with plant hormones in the medium that leads to cell division induction and plant regeneration (Pasternak et al., 2002; Wang et al., 2011; Rose et al., 2013). The importance of ROS in signaling at both local and systemic levels, with plant hormone webs, is now widely recognized (Mittler et al., 2011). High ROS levels for prolonged periods can of course be highly damaging to cellular components (Foyer and Noctor, 2011), however, regulating ROS levels after ROS bursts necessitates suitable homeostatic mechanisms for cell metabolism and development requirements.

The oxidative burst that accompanies protoplast isolation and early culture corresponds to the period of maximal cellular dedifferentiation (Zhao et al., 2001). Indeed, in animal cells, dedifferentiation (G_0_ to G_1_ transition) is regulated by redox controls that converge on cyclin D (Burch and Heintz, 2005), while in plant cells, moderate ROS levels up-regulate cyclin dependent kinase A1 activity and accelerate the cell cycle activation of differentiated leaf cells (Féher et al., 2008). Thus, an oxidative burst in protoplasts appears necessary to transition from G_0_ to G_1_. Although an oxidative burst seems to promote dedifferentiation, a reduced cellular environment is necessary for continued progression through the cell cycle. The status of redox couples in the cell connects environmental signals to the regulation of cell division, by controlling progression through the G_1_ to S phase checkpoint (Reichheld et al., 1999; Foyer and Noctor, 2005). Two of the most important redox couples in plant cells are the ascorbate-dehydroascorbate [ASC/DHA] and glutathione-glutathione disulphide [GSH/GSSG] couples, with direct evidence for both redox couples influencing the transition from G_1_ to S phase in plant cells (Liso et al., 1988; Cordoba-Pedregosa et al., 1996; Potters et al., 2000, 2004). Our findings that glutathione and ascorbate were predominantly reduced and abundant, shortly after the initiation of protoplast culture, suggests that the redox-signaling environment in cultured protoplasts is primed for progression through the cell cycle. This is in contrast to the slowly dividing cells of the root quiescent center, where ascorbate and glutathione are maintained at low levels and in a primarily oxidized form (Kerk and Feldman, 1995; Sanchez-Fernandez et al., 1997). The fact that ascorbate and glutathione were present in the reduced state, also implies that either the cellular machinery that recycles the oxidized forms of these antioxidants is highly active or alternatively, that these molecules do not participate in frontline ROS detoxification. The increasing expression of MDAR (specifically MDAR1) involved in the reduction of MHA coupled with the relatively unchanging expression of GSSG (GR1) and the low total activity of GR in protoplasts (maximum activity was ∼0.005 μmol⋅min^-1^⋅μg^-1^ of cells at 96 h) relative to the total concentration of glutathione (∼0.35 μmol⋅μg^-1^ of cells at 96 h) suggests that the latter alternative is more likely.

### Changes in Peroxisome Proliferation and Peroxisome-Localizsed Antioxidants in Relation to Cell Division Induction

There is a very large increase in peroxisome numbers and total peroxisome volume associated with the protoplast culture prior to the first cell division. Consistent with the increased peroxisome numbers and volume, transcript levels of most peroxisome genes involved in biogenesis and proliferation were upregulated during early protoplast culture (Supplementary Figure S1). This increased peroxisome number contributes to an increased antioxidant capacity with the increased numbers tightly inversely correlated with ROS levels. In cultured suspension cells there is only one regular doubling of peroxisomes at each cell cycle (Lingard et al., 2008). This may also be the case for dividing cells of the meristems in plants.

Peroxisomes participate in a variety of processes related to oxidative metabolism, and the expression of genes for peroxisome-localized antioxidants is influenced by both metabolic and environmental cues (León, 2008; Palma et al., 2009). Peroxisomes generate ROS, but can also rescue cells from the damaging effects of ROS. Accordingly, peroxisomes are the main site for renewal of cellular antioxidants and house an arsenal of antioxidant enzymes; they are also the sole location of the cells principal H_2_O_2_-degrading enzyme, CAT (Lopez-Huertas et al., 2000; Corpas et al., 2001). Total CAT activity and *CAT3* (but not *CAT1* or *CAT2*) gene expression increased during culture (**Figure 6C**). Interestingly, *CAT3* is also upregulated in response to oxidative stress in senescing tissues in *Arabidopsis* (Du et al., 2008). The other peroxisome-localized gene directly involved in the detoxification of ROS is *APX3*. However, the rate of increase in *APX3* expression after 48 h protoplast culture, when ROS levels decline most precipitously (**Figure 4B**) was only 62% of that for *CAT3* (**Figures 6B,C**). Moreover, the K_cat_/K_m_ values for plant CAT, which are in the range of 10^4^–10^5^ s^-1^⋅μM^-1^ (Havir and McHale, 1990), dwarf those of APX at around 10^-1^–10^0^ s^-1^⋅μM^-1^ (Lad et al., 2002). Because of a low affinity (K_m_) for H_2_O_2_, but high turnover rate (K_cat_), CAT is ideally suited to the removal of large amounts of ROS, as appears to be the case following an oxidative burst.

Peroxisomes respond to ROS generated in other intra- or extracellular locations, probably to protect the cell from excessive oxidative damage (Lopez-Huertas et al., 2000; Schrader and Fahimi, 2006), while over expression of CAT in mice extends their lifespan (Schriner et al., 2005). These data (**Figure 7**) suggest peroxisomes, and in particular CAT, play a key role in restoring homeostatic levels of ROS, whereas, ASC/DHA and GSH/GSSG redox couples appear to have a more modulating role as the protoplasts proceed toward cell cycle initiation. Homozygous knockout mutants of *PEX11a*, which is involved in regulating peroxisome numbers (Lingard and Trelease, 2006; Orth et al., 2007), and *CAT3* cause reduced cell division. This is consistent with peroxisomes having an important role in the restoration of ROS levels after the oxidative burst, establishing an optimum subcellular redox environment for plant cell cultures acquiring totipotency.

### Peroxisome Dynamics: Dual Functions in Inheritance and Redox Homeostasis?

The uniform dispersion of peroxisomes throughout the cytoplasm ensures unbiased partitioning of these now small and numerous organelles and facilitates peroxisomal functions. Transient clustering of peroxisomes near the developing cell plate in onion cells (Collings et al., 2003) and the classical association of peroxisomes with chloroplasts and mitochondria in photosynthesizing mesophyll cells suggests that peroxisomes do localize to specific sites when required. Interestingly, proteins responsible for peroxisome biogenesis and morphology are induced by ROS, and in both plant and animals cells peroxisomes proliferate in response to stress. Indeed, a number of studies in plants link peroxisome proliferation with stresses that perturb ROS homeostasis (Chang et al., 1999; Lopez-Huertas et al., 2000; del Rio et al., 2006). Whether peroxisomes proliferate simply because of stress, or instead, to enhance peroxisomal activities that protect against oxidative stress, is still unclear. However, the latter is consistent with the cellular physiology. The findings of this study and our previous work (Sheahan et al., 2004, 2007b) indicate that plant organelles use diverse partitioning strategies to ensure unbiased inheritance at cell division. These strategies, however, presumably evolved within the constraints of maintaining cellular function, such as modulating the redox environment during morphogenic responses to stress (Potters et al., 2007), a key factor facilitating totipotency in plants.

## Conflict of Interest Statement

The authors declare that the research was conducted in the absence of any commercial or financial relationships that could be construed as a potential conflict of interest.

## Footnotes

1 http://www.ncbi.nlm.nih.gov/tools/primer-blast/

2 http://imagej.nih.gov/ij/
