# Supplementary material for: Peroxisomes contribute to reactive oxygen species homeostasis and cell division induction in Arabidopsis protoplasts
Source: Front Plant Sci. 2015 Aug 26;6:658. doi: 10.3389/fpls.2015.00658 (PMC4549554; doi:10.3389/fpls.2015.00658)
Supplement: Supplementary file 1 [file Table_1.PDF]

Supplementary Table S1: Primers used in qPCR

Gene Name	AGI ID	Primers (5' - 3')
APX3	AT4G35000	TGGCATGGCACGATGCTGGA
		ACCACACCAGCAAGCTGATACAGG
BSMDR	AT1G49670	GCCAAACCGTGGCTGGTTTCT
		CCGGTGCTTTTACCCGAATGCAG
CAT1	AT1G20630	CATGCACAGGGATGAGGAGGTCAA
		GCTGCGGATTTCGTGCGTGA
CAT2	AT4G35090	CATGCACAGGGACGAGGAGGTTA
		GCGGATTTCATGCGTGATGCGT
CAT3	AT1G20620	TGTCCGCTGCGCTGAGAAAGT
		AGGATCGATCAGCCTGAGACCAGT
CSD3	AT5G18100	GGCGATAACAACGTCCGAGGCT
		TCAGCAACACCGTTTGATCCTGC
EMB2024	AT5G24400	GCGCTTTCACCGTCGTTGTCT
		CCAGGCGGAATTGGTACCTTGGA
GR1	AT3G24170	GGCCACCTGTTTTGCGAACACT
		AGCTGCATCAGGACCGCACA
GSTT1	AT5G41210	GGCAGGCTTAAGCTCTTTGAGAGTCAC
		TCCAGCTGCACCACGACGTA
MDAR1	AT3G52880	CCCTGAACCTTGGTGCATGCCT
		GTCGGCTTCCAATGTCCTCCCA
MDAR4	AT3G27820	GCTCACTGCATGGCACGTCTCT
		GCTGGTAAGTGGCTCCCGTCTT
UBQ10	AT4G05320	TGTTGCGTCTGCGTGGAGGT
		CGCTGCTGGTCCGGAGGAAT
