# Supplementary material for: Peroxisomes contribute to reactive oxygen species homeostasis and cell division induction in Arabidopsis protoplasts
Source: Front Plant Sci. 2015 Aug 26;6:658. doi: 10.3389/fpls.2015.00658 (PMC4549554; doi:10.3389/fpls.2015.00658)
Supplement: Supplementary file 2 [file Data_Sheet_1.PDF]

Supplementary Figure Legends

Figure S1: Heat map showing gene expression profiles of peroxisomes during protoplast culture. Expression profile of Arabidopsis peroxisomes genes involved in biogenesis and proliferation during culture of protoplasts over 96 h. Also included are genes involved in peroxisome fission machinery DRP3s and FIS1s. Regulation of expression is designated as fold-change relative to 0 h.

Figure S2: Quantification of peroxisome distribution. Clustering was defined manually, where three or more peroxisomes had touching faces in at least two optical sections, whereas cells with gross aggregation were defined as those having a large proportion of the peroxisome population sequestered in a localised region of cytoplasm.

Figure S3: Peroxisome proliferation in ROS detoxification (*Nicotiana tabacum*).
(A) Hydrogen peroxide production as measured in macerated leaves (source tissue for protoplast isolation) and in freshly prepared (0 h) protoplasts. (B) Hydrogen peroxide levels in cultured protoplasts decrease during culture and before most cells divide. (C) Cell division rate for cultured protoplasts. Cell division occurs maximally after 48 h culture. (D) Correlation between peroxisome number and ROS level. Decreasing ROS levels correlate well with increasing peroxisome numbers ($R^2 = 0.99$). Values are means ± se with $n \geq 3$.

Figure S4: Redox Sensor Red (RSR) staining during protoplast culture (*Nicotiana tabacum*). Nicotiana protoplasts were incubated for 6 h in RSR after each 24 h period. Proportion of colocalisation was determined by proportion of RSR stained mitochondria [by overlay, also see Fig. 5E (0 h)] relative to total RSR staining in a cell. There are increasingly fewer RSR stained mitochondria with increased incubation time. Values are means ± s.e.m with n ≥ 12 . For more information on the cell biology of mitochondria in protoplast culture see Sheahan et al. (2004,2005).

Figure S5: The effect of the cytoskeletal inhibitors Latrunculin B and oryzalin on peroxisome proliferation, size and total volume during Arabidopsis protoplast culture. Values are means ± s.e.m with n = 3.

Peroxisome Genes

Name	Culture Duration (h) 24	48	72	96
PEX6	****	*****	*****	*****
PEX3-1	****	****	*****	*****
PEX1	****	****	***	***
PEX2	****	****	***	***
PEX5	***	****	****	***
PEX14	***	****	***	****
PEX16	***	****	***	****
PEX4	***	***	***	***
PEX10	***	***	***	***
PEX19-1	***	***	***	***
DRP3B	***	***	***	***
DRP3A	***	***	***	**
PEX11b	***	-	-	*
PEX17	**	****	****	****
PEX7	**	***	***	***
PEX12	**	***	***	***
PEX3-2	**	**	*	*
PEX19-2	**	**	*	*
PEX11a	*	**	**	***
PEX11e	*	**	**	*
PEX13	*	**	*	**
PEX11c	*	*	*	*
PEX22	*	*	*	*
FIS1B	*	*	*	*
PEX11d	*	*	*	-
FIS1A	*	*	-	-

Legend Relative Fold-change	
< 1	-
≥ 1	*
≥ 2	**
≥ 3	***
≥ 4	****
≥ 5	*****

Supplementary Figure 1

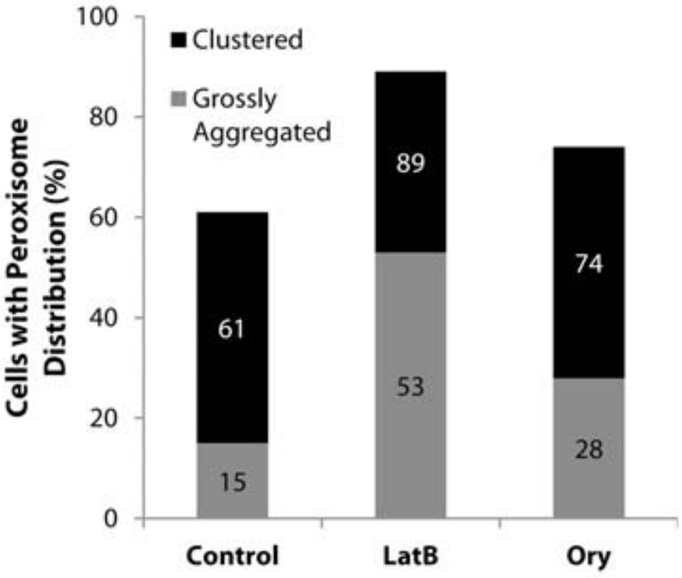

Supplementary Figure 2

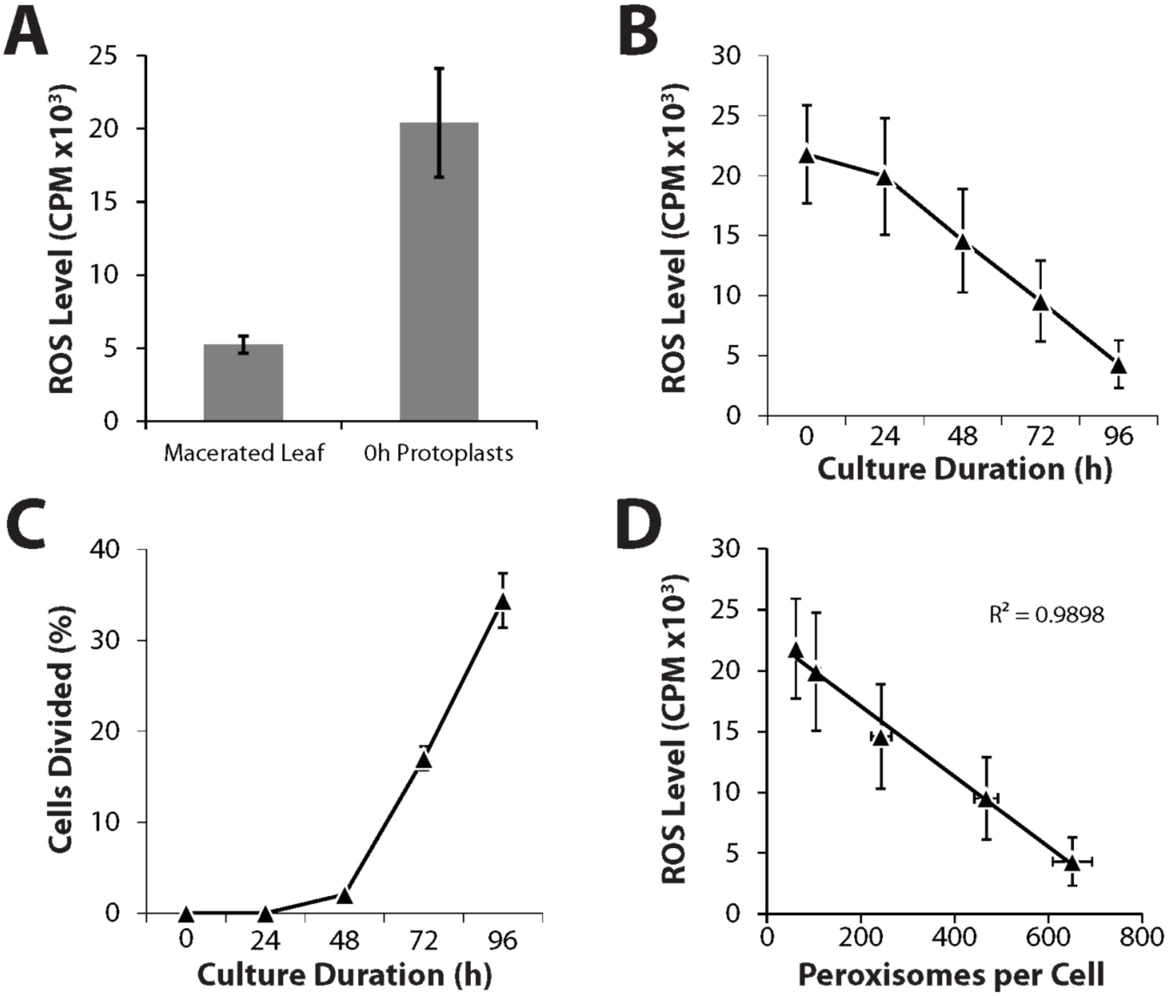

Supplementary Figure 3

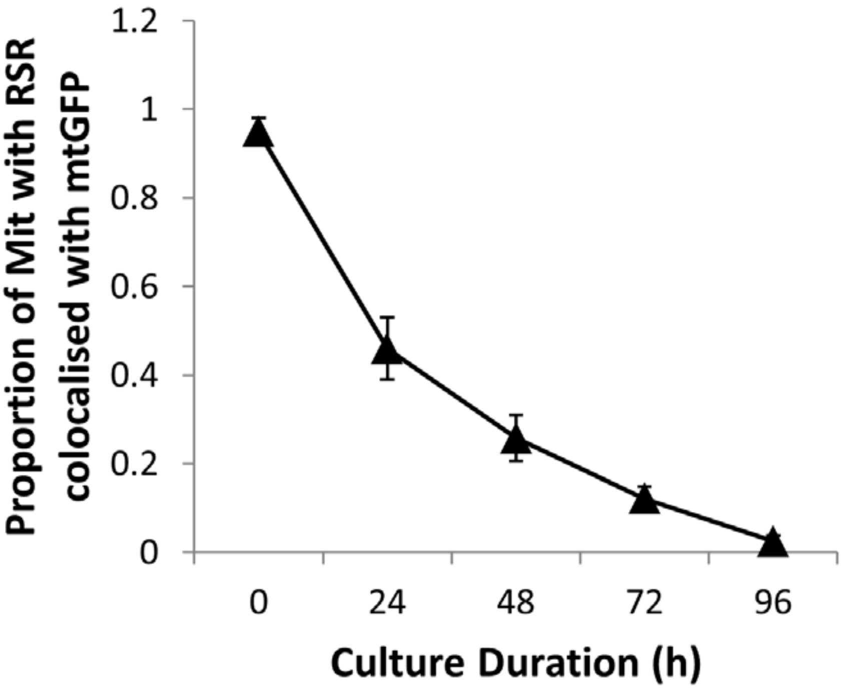

Supplementary Figure 4

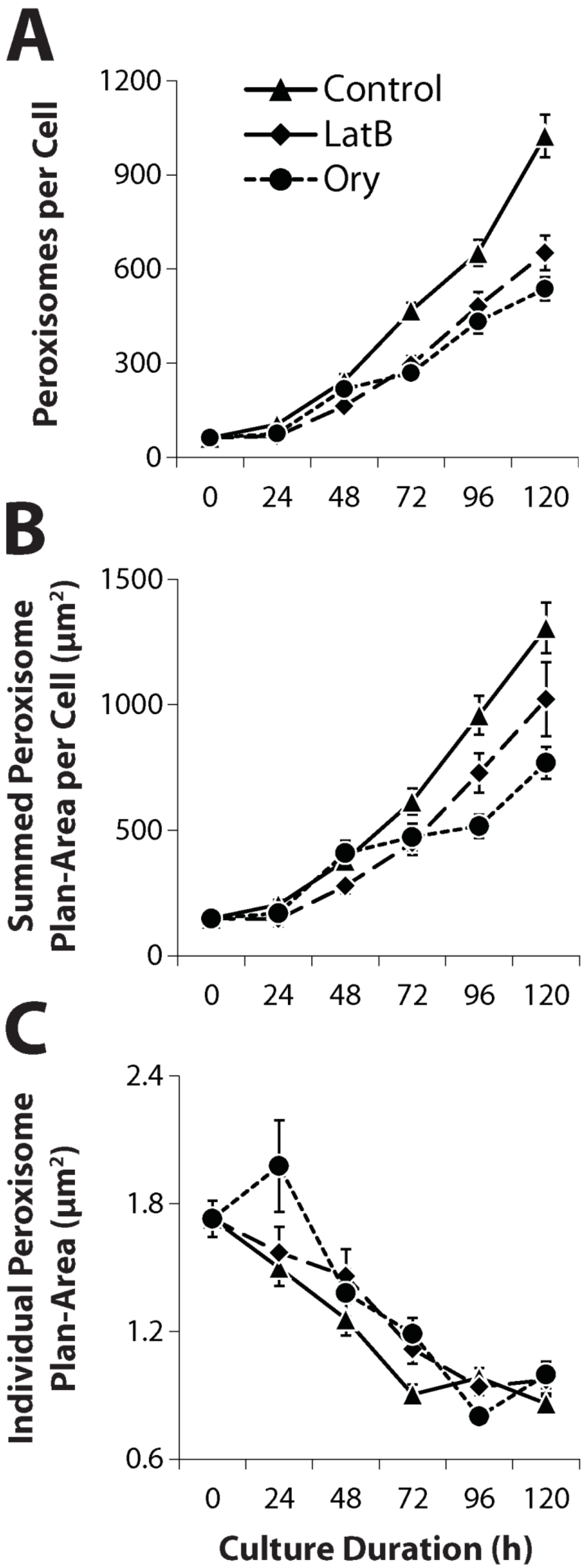

Supplementary Figure 5
